# Postpartum thrombotic microangiopathy revealed as atypical hemolytic uremic syndrome successfully treated with eculizumab: a case report

**DOI:** 10.1186/1752-1947-8-307

**Published:** 2014-09-14

**Authors:** Andreas Kourouklaris, Kyriakos Ioannou, Ioannis Athanasiou, Alexia Panagidou, Kiproulla Demetriou, Michalis Zavros

**Affiliations:** 1 Department of Nephrology, Nicosia General Hospital, B1, Strovolos, Nicosia, Cyprus

## Abstract

**Introduction:**

Differential diagnosis of thrombotic microangiopathies can be difficult. Atypical hemolytic uremic syndrome is a rare, life-threatening disease caused by uncontrolled chronic activation of alternative complement pathway, resulting in microvascular thrombosis, organ ischemia and damage. Prognosis is poor: up to 65 percent of patients require dialysis or have kidney damage of varying severity or die despite plasma exchange/plasma infusion treatment.

**Case presentation:**

We describe the case of a 23-year-old woman of Hellenic origin who, after a preeclampsia-induced premature delivery, developed thrombotic microangiopathy with renal failure, tonicoclonic seizures, anasarca edema and hypertension. Intensive plasma exchange was initiated twice daily, in parallel to dialysis for one month. Three months later, our patient was discharged with nondialysis-dependent renal failure and without signs of hemolysis. Three months after discharge our patient was readmitted with cardiomyopathy (left ventricular ejection fraction of 25 percent) and signs and symptoms of thrombotic microangiopathy. Our patient was diagnosed with atypical hemolytic uremic syndrome and was started on eculizumab (a complement inhibitor), which improved clinical and laboratory parameters. However, a transient pause in treatment resulted in thrombotic microangiopathy relapse, which was rapidly blocked with reintroduction of eculizumab treatment. During long-term eculizumab treatment, thrombotic microangiopathy manifestations were inhibited and renal and cardiac function restored, with no need for other invasive treatments.

**Conclusions:**

Establishing the diagnosis of atypical hemolytic uremic syndrome in patients presenting with thrombotic microangiopathy is challenging since common symptoms are shared with other conditions like Shiga toxin-producing *Escherichia coli* hemolytic uremic syndrome and thrombotic thrombocytopenic purpura. The described case illustrates the complexity and importance of rapid diagnosis in a rare disease and the need for appropriate and specific treatment for best long-term outcomes.

## Introduction

Thrombotic microangiopathy (TMA) can be a manifestation of several medical conditions, like connective tissue diseases, malignancy and posttransplantation. However, TMA manifestations dominate and characterize diseases like thrombotic thrombocytopenic purpura (TTP), atypical hemolytic uremic syndrome (aHUS) and Shiga toxin-producing *Escherichia coli* hemolytic uremic syndrome (STEC-HUS), thus making differential diagnosis of TMA difficult. Both STEC-HUS and aHUS are characterized by hemolytic anemia, thrombocytopenia and organ failure. STEC-HUS is more common in children and is associated with a prior infection from STEC and presence of Shiga toxin causing endothelial damage and complement activation [1]. aHUS is characterized by genetic hyperactivation of the alternative complement pathway and can present in both children and adults [2-4]. A genetic mutation in complement regulatory genes has been identified in approximately 60 percent of patients with aHUS [3,5,6]. Dysregulation of the complement system leads to endothelial, neutrophil and platelet activation causing TMA associated with hemolytic anemia and thrombocytopenia, which in turn may cause severe organ damage in multiple vital organs [7]. TTP is another form of TMA associated with severe ADAMTS13 deficiency. ADAMTS13 is an enzyme that cleaves the ultralarge von Willebrand factor multimers that can form in plasma during shear stress causing platelet aggregation and thrombosis. Management of TMA often involves plasma exchange and/or plasma infusion (PE/PI) in an attempt to remove mutant forms and restore functional proteins. It seems to be effective in the management of TTP [8], however, outcomes are still poor in aHUS and a large percentage of patients still progresses to end-stage renal disease (ESRD) or die at first clinical manifestation of TMA [3,5,6,9]. Eculizumab is a humanized monoclonal antibody that binds to complement component C5 inhibiting its cleavage to C5a and C5b, inhibiting complement-mediated TMA in patients with aHUS [10]. Since its introduction in aHUS treatment, eculizumab has exhibited improved outcomes compared to plasma exchange (PE) in prospective clinical trials and in several published cases [10-15]. Eculizumab has been approved for the treatment of aHUS and is well tolerated [5,12,16]. We present the case of a young woman of Hellenic origin who presented with signs and symptoms of TMA following a preeclampsia-induced premature delivery. Our patient was initially managed with PE and dialysis, but TMA multiorgan manifestations persisted and improved only upon chronic eculizumab treatment.

## Case presentation

A 31-week-pregnant young woman of Hellenic origin (age 23), free of previous medical history, was admitted in January 2011 for an urgent cesarean section due to preeclampsia presenting with nephrotic-range proteinuria (7gr/24hrs), increased blood pressure (180/100mmHg) and edema. Five days post-cesarean section, she presented hemolytic anemia, thrombocytopenia, renal impairment, tonicoclonic seizures, and hypertensive crisis. Schistocytes were detected on her peripheral blood smear, while elevated lactate dehydrogenase (LDH) 3254IU/L (laboratory normal range (LNR): 208 to 408IU/L), total bilirubin (TBIL) 4.2mg/dL (LNR: 0.3 to 1.2mg/dL) and serum creatinine 4.9mg/dL (LNR: 0.67 to 1.17mg/dL), as well as decreased platelet count (PLT: 40×10^9^/L) (LNR: 150 to 480×10^9^/L) were recorded. Intensive PE treatment was initiated (twice daily for 22 days), in parallel with dialysis for volume and uremia control.

**Figure 1:**
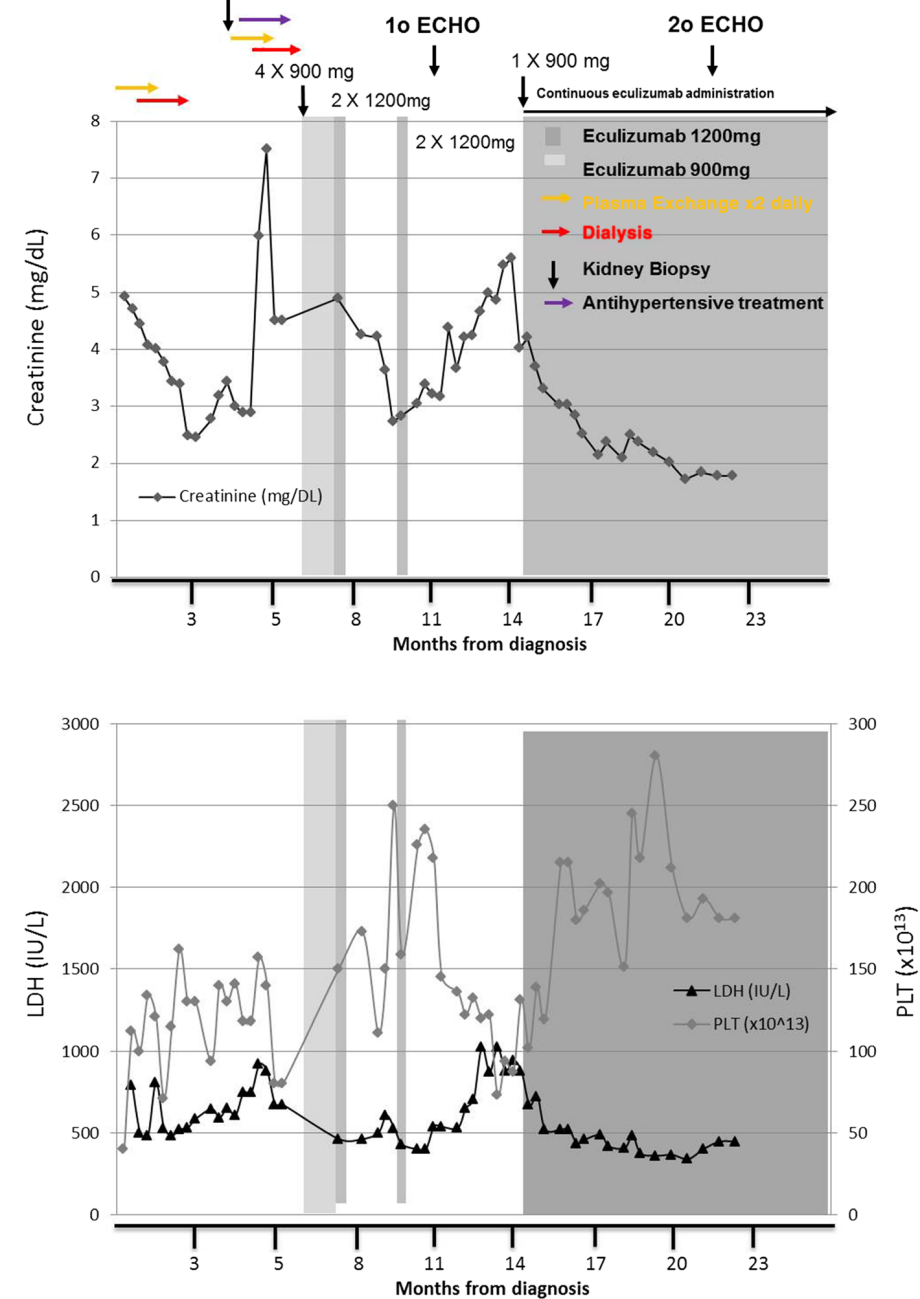
**Response to eculizumab treatment in a young woman of Hellenic origin who was diagnosed with atypical hemolytic uremic syndrome following a preeclampsia-induced premature delivery.** At admission, laboratory results indicated thrombotic microangiopathy, acute kidney failure and thrombocytopenia; creatinine level 4.9mg/dL; lactate dehydrogenase 3254IU/L; platelets 40×10^9^/L, with the presence of schistocytes. Plasmapheresis and dialysis managed to temporarily correct clinical indices, however, following deterioration of her clinical status, our patient was started on eculizumab, 900mg per week, in July 2011. Her clinical condition started to improve after the first administration of eculizumab. However, due to drug accessibility, the complete dosing schedule of eculizumab could not be maintained and our patient presented with pneumonia and cardiac failure and was admitted to the intensive care unit. Due to the persistence of her clinical condition (thrombocytopenia; platelets 73×10^9^/L, renal failure; creatinine 4.85mg/dL, microangiopathy; lactate dehydrogenase 1024IU/L), eculizumab was reinstated starting at 900mg followed by 1200mg fortnightly. Clinical and laboratory symptoms of thrombotic microangiopathy started to decline and our patient achieved normal renal function after administration of the first doses of eculizumab. Our patient remains on 1200mg eculizumab administered every two weeks and is free of any symptoms of thrombotic microangiopathy.

Following PE and dialysis her clinical condition transiently improved (serum creatinine: 2.8mg/dL, LDH: 605IU/L, PLT: 141×10^9^/L). Approximately four months later, in June 2011, her renal function deteriorated again (serum creatinine 8mg/dL) and laboratory investigations showed hemolytic anemia, thrombocytopenia (PLT: 80×10^9^/L), a negative Coombs test and detectable ADAMTS13 activity excluding TTP. Stool samples could not detect *Escherichia coli* O157:H7/O104:H4 or Shiga toxin, excluding STEC-HUS. A kidney biopsy was performed, which revealed evidence of progressing TMA with excessive neutrophil infiltrations, capillary loop blockade occlusions and abnormal capillary wall thickening. PE and dialysis were restarted; however, excessive vomiting and hemorrhagic diarrhea were added to the TMA clinical manifestations. Our patient was then started on eculizumab in July 2011, after she was vaccinated against *Neisseria menigitidis*. Treatment included 900mg weekly eculizumab for four weeks, followed by one dose of 1200mg. A six-week interruption of eculizumab treatment occurred due to drug accessibility. During this period, our patient was managed with dialysis and eculizumab was reinstated at 1200mg every two weeks in October 2011. However, following two doses of eculizumab, our patient presented with pneumonia (tests for *Streptococcus pneumoniae* and *Haemophilus influenza* were negative), cardiac failure (left ventricular ejection fraction (LVEF) via transthoracic echocardiography: 35 to 40 percent; pulmonary hypertension: right ventricular systolic pressure (RVSP): 47.1mmHg) and hypertensive crisis. She was treated with antibiotics, diuretics and antihypertensives, and eculizumab treatment was discontinued again due to our patient’s decision.During this period, her anemia worsened (hemoglobin 7.5g/dL), her LDH and TBIL increased two-fold (428mg/dL to 1023mg/dL and 0.7mg/dL to 1.9mg/dL respectively), her renal function was compromised (creatinine 5.5mg/dL) and schistocytes were observed on the peripheral blood smear. Hence, a decision was made to reintroduce chronic eculizumab treatment, starting with one dose of 900mg, which was followed by 1200mg every two weeks as a maintenance dose. Her creatinine and LDH levels decreased rapidly to normal ranges while her thrombocytopenia was also reversed (Figure 1). Until her last follow-up visit on May 2013, our patient remained on chronic eculizumab treatment, free of any TMA-related complications (creatinine level, 1.5m/dL). No PE/PI, no dialysis and no blood transfusions were necessary to maintain normal organ function. Her serum creatinine levels were stable at 1.5mg/dL, while her LDH levels and PLT remained stable within normal ranges. A follow-up transthoracic echocardiography was performed in December 2012, and another in January 2013 where her cardiac function was stable; her cardiac function was improved (LVEF: 45 to 50 percent) and her pulmonary pressure was normalized (RVSP <35mmHg).

## Discussion

Differential diagnosis of TMA presenting in an adult can be very challenging. Similar clinical presentation is shared by the three most common causes of TMA; aHUS, TTP and STEC-HUS. Co-existing conditions like connective tissue disorders, drug use after transplantation, humoral rejection or malignancy can also complicate the picture. It has been suggested that identifying severe ADAMTS13 protease deficiency (<5 to 10 percent) is a valid way to differentiate TTP from aHUS [8,17]. In cases where ADAMTS13 activity estimation is not available, Coppo *et al*. have shown that low platelet count (<30×10^9^/L) and serum creatinine below 2.26mg/dL is highly associated with severe ADAMTS13 deficiency and TTP [18]. Additionally, postpartum TMA is mostly associated with aHUS, while TMA during pregnancy is mostly related severe ADAMTS13 deficiency [19]. STEC-HUS can occur in adults, but requires an identification of either the bacteria producing Shiga toxin or the presence of Shiga toxin. Complement measurements may not always be useful as a rather large percentage (64 percent) of patients with aHUS, have normal complement levels of C3 [20]. Undertaking genetic screening will not provide an answer rapidly enough for immediate diagnosis. In our case, genetic testing was not accessible and CH50 was not measured. However, C3 levels were constantly low, indicating alternative complement pathway activation and, on relapse, C3 levels showed further decrease. Furthermore, her serum creatinine at presentation was 4.9mg/dL and PLT 40×10^9^/L, which did not favor the diagnosis of TTP, while no Shiga toxin or Shiga toxin-producing bacteria were found, thus excluding STEC-HUS. Therefore, the diagnosis of aHUS seemed the most probable in this case.

PE/PI has been extensively used to manage TMAs [3]. However, despite optimum PE/PI treatment, more than one-third of patients with aHUS become refractory to PE, develop ESRD or die [11,16]. Thus identifying patients with aHUS is important for the best long-term patient outcomes. Eculizumab has been associated with rapid inhibition of complement activity and higher long-term efficacy in blocking systemic TMA and restoring organ function, compared to PE in patients with aHUS [15,21,22].

Intensive plasma exchange and dialysis improved our patient’s condition transiently but failed to preserve renal function. Our patient’s condition deteriorated further with symptoms resulting from hemolytic anemia and thrombocytopenia, while cardiac and lung function were also affected. Introduction of eculizumab rapidly inhibited TMA, improved renal function and restored normal PLT. However, discontinuation of treatment, which in the present case occurred twice, may have hampered the corrective effects of eculizumab. Previous reports have shown that dose reduction or discontinuation of eculizumab treatment lead to TMA relapse, which may lead to rapid deterioration of vital organs [23-25]. Upon restarting eculizumab, the induction dose of 900mg/week for four weeks, followed by 1200mg on the fifth week is also recommended for efficacious complement blockade. In the present case, the two doses given after the sustained treatment break of six weeks may not have been adequate to block complement, as there was further progression of renal failure, thrombocytopenia and elevation of LDH. Our patient developed cardiomyopathy (LVEF reduced to 25 percent) and pulmonary hypertension (RVSP of 47.1mmHg) that could be explained by stunned myocardium. Diuresis for a few days improved her heart failure symptoms, but echo findings did not improve significantly in follow-up echo studies. Reintroduction of chronic eculizumab treatment led to TMA control, followed by remission and dramatic improvement not only of renal function and blood parameters, but also of echo findings. The last echocardiographic examination, six months post-relapse episode, showed significant improvement of cardiac function (LVEF of 45 percent) and reversal of pulmonary hypertension, suggesting that TMA could have contributed to the impaired cardiac function.

Uncontrolled chronic activation of the alternative complement pathway is the underlying cause of systemic microvascular thrombosis, leading to organ ischemia and damage. Eculizumab appears as the treatment of choice for long-term management of aHUS. An increasing body of evidence, both from clinical investigations and case reports of patients with variable medical history, indicate that eculizumab is effective in blocking further progression of TMA in patients with aHUS [5,13,14].

In the present case of aHUS complicated with multiple organ damage, sustained eculizumab treatment managed to rapidly block complement activation, reverse renal impairment and longitudinally attenuate organ ischemia and damage. Therefore, it is of great importance to identify aHUS patients early and rapidly initiate, but also maintain, eculizumab treatment to avoid unnecessary complement reactivation that could lead to irreversible and possibly life-threatening organ damage.

## Conclusions

The present report described a rare case of aHUS complicated with multiple organ damage. Establishing the diagnosis of aHUS in patients presenting with TMA is challenging since common symptoms are shared with other conditions like STEC-HUS and TTP. In addition, although ADAMTS13 activity is key factor for the differential diagnosis of TMAs, if not available, other indicators, such as platelet and creatinine levels should be considered, especially in cases of pregnancy-related TMA. Finally, sustained eculizumab treatment has managed in our case to rapidly block complement activation, reverse renal impairment and longitudinally attenuate organ ischemia and damage, while PE treatment failed to reverse TMA. Therefore, the rapid identification of aHUS patients along with initiation and maintenance of proper treatment are essential for a favorable outcome in this life-threatening, rare condition.

## Consent

Written informed consent was obtained from the patient for publication of this case report and any accompanying image. A copy of the written consent is available for review by the Editor-in-Chief of this journal.

## Abbreviations

aHUS: atypical hemolytic uremic syndrome; C3: complement component 3; C5: complement component 5; ESRD: end-stage renal disease; LDH: lactate dehydrogenase; LNR: laboratory normal range; LVEF: left ventricular ejection fraction; PE: plasma exchange; PE/PI: plasma exchange/plasma infusion; PLT: platelet count; RVSP: right ventricular systolic pressure; STEC-HUS: Shiga toxin-producing *Escherichia coli* hemolytic uremic syndrome; TBIL: total bilirubin; TMA: thrombotic microangiopathy; TTP: thrombotic thrombocytopenic purpura.

## Competing interests

All authors declare that they have no competing interests.

## Authors’ contributions

AK made substantial contribution to conception and design, acquisition of data, analysis and interpretation of data. KI made substantial contribution to conception and design, acquisition of data, analysis and interpretation of data. IA made substantial contribution to conception and design, acquisition of data, analysis and interpretation of data. AP made substantial contribution to acquisition of data, analysis and interpretation of data. KD made substantial contribution to acquisition of data, analysis and interpretation of data. MZ made substantial contribution to conception and design, acquisition of data, analysis and interpretation of data and gave final approval of the version to be published. All authors read and approved the final manuscript.
